# Conditional and Specific Cell Ablation in the Marine Annelid *Platynereis dumerilii*

**DOI:** 10.1371/journal.pone.0075811

**Published:** 2013-09-24

**Authors:** Vinoth Babu Veedin-Rajan, Ruth M. Fischer, Florian Raible, Kristin Tessmar-Raible

**Affiliations:** 1 Max F. Perutz Laboratories, University of Vienna, Vienna, Austria; 2 Research Platform “Marine Rhythms of Life,” Vienna, Austria; University of Michigan, United States of America

## Abstract

The marine annelid Platynereis dumerilii has become a model system for evo-devo, neurobiology and marine biology. The functional assessment of its cell types, however, has so far been very limited. Here we report on the establishment of a generally applicable, cell type specific ablation technique to overcome this restriction. Using a transgenic strain expressing the bacterial enzyme nitroreductase (ntr) under the control of the worm’s r-opsin1 locus, we show that the demarcated photoreceptor cells can be specifically ablated by the addition of the prodrug metronidazole (mtz). TUNEL staining indicates that ntr expressing cells undergo apoptotic cell death. As we used a transgenic strain co-expressing ntr with enhanced green fluorescent protein (egfp) coding sequence, we were able to validate the ablation of photoreceptors not only in fixed tissue, using r-opsin1 riboprobes, but also by monitoring eGFP+ cells in live animals. The specificity of the ablation was demonstrated by the normal presence of the eye pigment cells, as well as of neuronal markers expressed in other cells of the brain, such as phc2, tyrosine hydroxylase and brn1/2/4. Additional analyses of the position of DAPI stained nuclei, the brain’s overall neuronal scaffold, as well as the positions and projections of serotonergic neurons further confirmed that mtz treatment did not induce general abnormalities in the worm’s brain. As the prodrug is administered by adding it to the water, targeted ablation of specific cell types can be achieved throughout the life of the animal. We show that ablation conditions need to be adjusted to the size of the worms, likely due to differences in the penetration of the prodrug, and establish ablation conditions for worms containing 10 to 55 segments. Our results establish mtz/ntr mediated conditional cell ablation as a powerful functional tool in Platynereis.

## Introduction

The marine bristle worm *Platynereisdumerilii* has become a reference system in the fields of evolution and development, as well as in comparative neuroscience. It has retained a more ancestral-type gene repertoire than conventional invertebrate molecular model systems [1], a feature that facilitates its comparison with vertebrates. Studies comparing the bristle worm’s nervous system and the vertebrate forebrain [2], the hypothalamus [3] and eye [4] reveal that key vertebrate brain cell types have correlates in the worm. However, all these comparisons have relied largely on gene expression analyses [5,6]. The functional importance of the respective cell types, however, has so far usually been inferred from other, functionally more established model systems. Although *Platynereis* cells can be specifically ablated by cold laser nanosurgery [7], this technique still requires manipulation of individual specimens and manual targeting of each cell, and therefore is not easily amenable to higher numbers of cells and worms.

Nitroreductase (ntr)-mediated, spatially and temporally controlled cell ablation, has been pioneered in zebrafish, and since used as a functional tool for the analyses of various tissues, such as pancreatic, heart and germ cells, as well as neuron types [8-10]. For this technique, the coding sequence for the bacterial enzyme Nitroreductase (Ntr), typically fused to a fluorescent protein for visualization, is expressed under the control of a cell type specific enhancer. By electrochemical reduction, Ntr efficiently converts the prodrug metronidazole (mtz) into a highly potent DNA interstrand cross-linking agent, which subsequently causes cell death. In zebrafish, Ntr/mtz-mediated cell death is confined to the *ntr* expressing cells, whereas the adjacent bystander cells are unaffected [8].

Here we report on the establishment of space and time controlled Ntr-mediated cell ablation in the marine bristle worm *Platynereisdumerilii* . For establishment, we used a transgenic strain that expresses both *egfp* and *ntr*, under the control of the recently described *r-opsin1* enhancer specifically in the worm’s *r-opsin1*+ photoreceptor cells (PRCs) [11]. Administration of mtz to the seawater leads to the specific ablation of all *r-opsin1*+ photoreceptors, but does not affect directly adjacent cell types, such as the pigment cells of the *Platynereis* adult eyes or other neuronal cell types in the worms’ brains, including their projections. Using this tool, we were able to ablate the *r-opsin1+* photoreceptors from a broad range of stages. Our work establishes the ntr/mtz system as a powerful technique to determine the functional requirement of specific cell types throughout the life of the bristle worm.

## Material and Methods

### Ethics statement

All animal work was conducted according to Austrian and European guidelines for animal research.

### Platynereis culture

*Platynereisdumerilii* were raised and bred in MFPL marine facility according to established protocols [12].

### Generation of r-opsin1::eGFP-F2A-NTR construct

The bacterial *nitroreductase* was PCR amplified from pNTR-EGFP (gift of Lazaro Centanin from Jochen Wittbrodt’s lab) adding the ribosomal skip site F2A, a stop codon, a BamHI restriction site at 5’ and a XhoI restriction site at 3’ using the following primers:

NTR-F2A-BamHI-fwd: GGATCCGTGAAACAGACTTTGAATTTTGACCTTCTCAAGTTGGCGGGAGACGTGGAGTCCAACCCAGGGCCCAAGCTTATGGATATCATTTCTGTCGCCTT; NTR-Stop-XhoI-rev: GTCACTCGAGGAGCTCCACCGCGGTGACTAGTAGTATCGATACGTCGACTTACACTTCGGTTAAGGTGATGTTTTG

**Figure 1:**
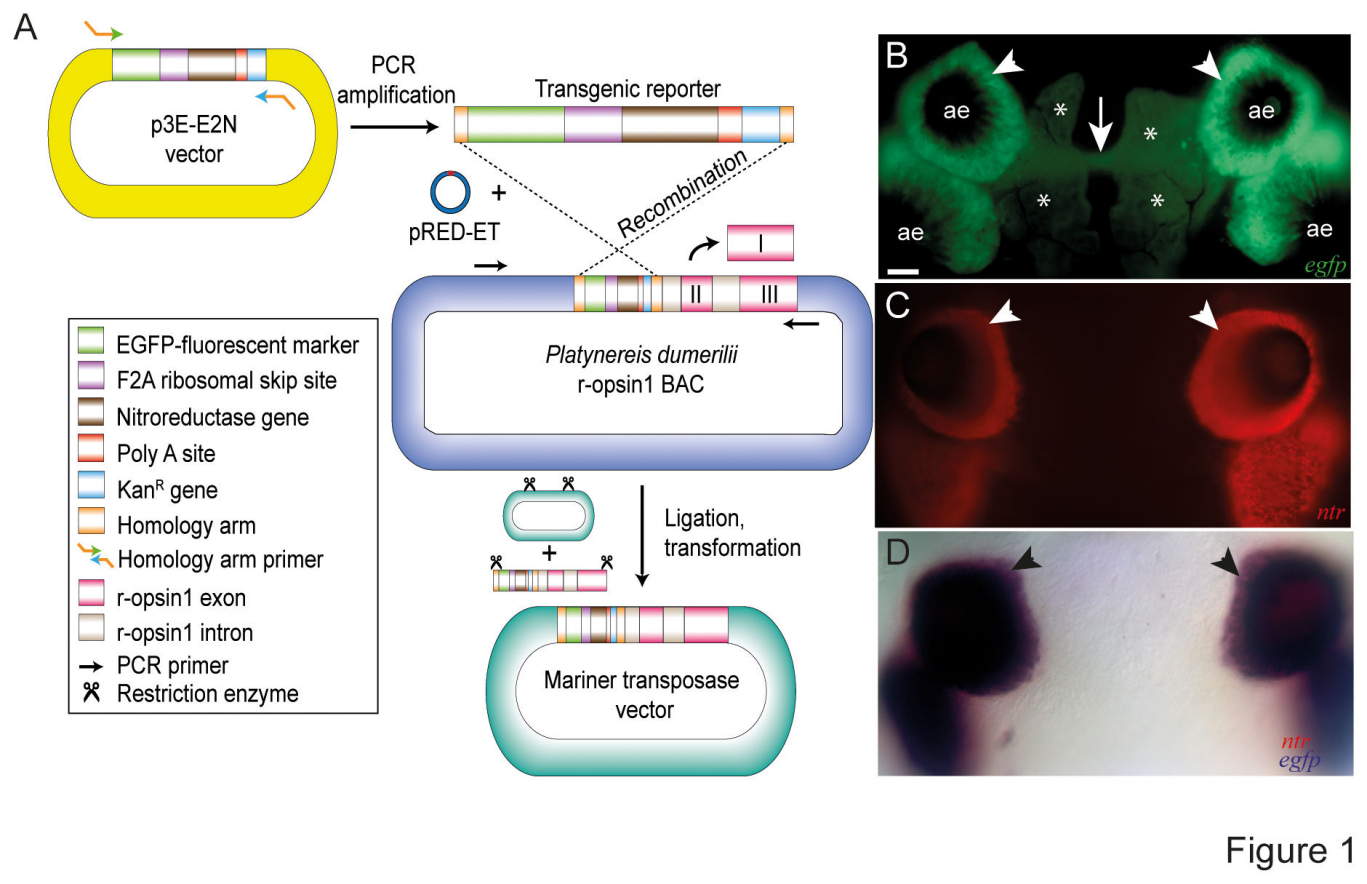
Construction and expression of the *r-opsin1::egfp-f2a-ntr transgene*. (A) Schematized generation of the *r-opsin1::egfp-f2a-ntr* construct. The *egfp-f2a-ntr* cassette was recombined into the *Platynereis* *r-opsin1* locus by homologous recombination. 8kbps of the surrounding genomic locus plus cassette were PCR amplified and subcloned into the mariner transposon vector used for transgenesis. (B-D) Co-expression of *egfp* and *ntr* in the adult eye photoreceptors of *r-opsin1::egfp-f2a-ntr* stable transgenic worms. (B) eGFP fluorescence demarcating the adult eye photoreceptors and their projections in stable *r-opsin1::egfp-f2a-ntr* transgenic worms. (C) Expression of *nitroreductase* (red) in the same cell type as visualized by whole mount in situ hybridization (WMISH) using *nitroreductase* antisense riboprobe. (D) Co-staining with *nitroreductase* (detected in red) and *egfp* (detected in blue) riboprobes results in purple color, indicative of faithful co-expression of both genes in the adult eye photoreceptors. ae- adult eyes; arrowheads point at expressing cells; arrow points at axonal projection of PRCs; asterisks- head pigment cells which show autofluorescence in the channel used for eGFP documentation. Scale bar: 20µm.

The fragment was cloned into the p3E-EGFPpA vector [13] at the BglII and XhoI sites. The cassette containing eGFP-F2A-NTR was subsequently PCR amplified using primers:

r-opsin1_upperGFPfusion: GAAAGGTCAGCCTTCTGTCGCCTACAACCACCAGCTTACCATGTCTCGGTCAGAGGATCCACCGGTCGCCACCATGGTGAGCAAGGGCGAGGAG

r-opsin1_lowerE2N: GGAAATTTTAAGGTTACATTTCAGCAAATAAGTGGGAAGAAACAAAGCGAAAAGACTTACGGAAAAAACCTCCCACACCTCCCC

and recombined to the first exon of a previously identified BAC containing the *r-opsin1* locus [1] using an established protocol for BAC homologous recombination [14]. An 8kbp fragment of the locus was PCR amplified and subcloned into pMosSce^frkt318^ as described in reference [11] (Figure 1A)

### Generation of r-opsin1::eGFP-F2A-NTR transgenic worms

The generation of transgenic worms is described in reference [11].

### Whole mount in situ hybridization

Co-localization and levels of *ntr* and *eGFP* transcripts in the transgenic animals were detected by using an established double fluorescent whole mount *in situ* hybridization protocol [11] with minor modifications. In brief: 20-35 segmented premature adult worms of the required genotype/ condition were fixed in 4% PFA/PTW with subsequent methanol washes. Fluorescein-labeled riboprobes were added along with the DIG-labeled riboprobes to the specimens and hybridized overnight at 65°C. Specimens were washed with SSCT at different concentrations, incubated for 1-2hrs in 5% sheep serum/1xPTW followed by incubation with anti-fluorescein-AP F_ab_ fragments (Roche) in a 1:1000 dilution in 2.5% sheep serum/1xPTW for overnight at 4°C. Subsequently, specimens were washed in 1xPTW for 4x15min and equilibrated in 0.1MTris/Hcl (pH 8.2) for 15min. Fast red staining reactions were performed using Fast Red tablets (Roche) dissolved in 0.1M TrisHCl (pH 8.2) according to manufacturer’s recommendations. After staining, specimens were washed twice in 1xPTW to stop staining and incubated in 0.1M glycine/HCl (pH2.2)/0.1% Tween 20 for 30min to inactivate the alkaline phosphatase. After washing 4x in 1xPTW, specimens were blocked for 1h in 5% sheep serum /1xPTW followed by incubation with anti-DIG-AP F_ab_ fragment (Roche) in a 1:2000 dilution in 2.5% sheep serum /1xPTW for overnight at 4°C. Specimens were washed 5x10min in 1xPTW and NBT-BCIP staining was performed.

### Metronidazole treatment

Metronidazole (mtz, Sigma, catalog no. M1547) was dissolved in 0.2% DMSO in artificial seawater with vigorous shaking and protected from ambient light. Artificial seawater (ASW) was prepared in accordance with ref [12]. : 10l ASW contained 300 g of Tropic Marine sea salt and 10 ml of each of the following 5 stock solutions. Stock solution 4 should be added after sterilization. Stock solution 1: 2g Na_2_-EDTA and 20 mg FeCl_3_ in 100 ml of distilled water. Stock solution 2: 200 mg H_3_BO_4_ and 20 mg Na _2_MoO _4_ in 50 ml of distilled water are added to 50 ml containing 230 mg ZnSO _4_, 65 mg MnSO_4_, 0.6 mg CoSO_4_, 0.1 mg CuSO _4_. Stock solution 3: 2.2 g KBr, 2 mg KI, 0.6 mg LiCl, 6 mg RbCl, 380 mg SrCl_2_, 3 mg AlCl_3_ consecutively dissolved in 100 ml of distilled water. Stock solution 4: 10 g NaNO_3_ and 2 g Na_2_HPO_4_ in 100 ml of distilled water. Stock solution 5: 2 g Sodiumsilicate in 100 ml of distilled water

We tested concentrations between 7-25mM mtz. Worms containing 10-20, 25-35 and 45-55 segments were treated with freshly prepared mtz or DMSO control dilutions, respectively, at 18±1°C in the dark for the time indicated. After treatment, the mtz containing or DMSO control medium was replaced by several washes with artificial seawater. Worms were subsequently maintained under normal culture conditions (50% ASW, 50% NSW) [12].

### Cryosectioning

Ablated and control adult *Platynereis* worms (45-55 segmented) were anesthetized in 50:50 7.5M MgCl_2_: Natural Sea Water (NSW) and subsequently decapitated. The heads were fixed with 4% PFA/PTW for 20min, subsequently incubated in 15% sucrose/phosphate-buffered saline (PBS) for two hours and in 30% sucrose/PBS overnight at 4°C. We used Tissue-Tek (O.C.T. Compound) to embed the specimens in molds and froze them in dry ice. A MicromHM500 OM Cryostat was used for obtaining 10µm thick sections, which were collected on cryosection-optimized coated glass slides (Superfrost plus, MENZEL-GLASER). The sections were stored immediately at -20°C.

### Immunohistochemistry

Tissue sections were re-hydrated for 2x5 min in 1xPTW and immediately blocked in 5% (vol/vol) sheep serum in 1xPTW for 30 min at room temperature. Polyclonal rabbit anti-5-HT serotonin antiserum (1:1000 dilution, Immunostar product No. 20080) and monoclonal mouse anti-acetylated tubulin antibody (1:250 dilution, Sigma Aldrich cat. no. T6793) were diluted in PBS/ 5% sheep serum, and incubated with the sections overnight at 4°C in a wet box covered with parafilm strips. Slides were washed 4x5mins in 1xPTW and further incubated with Cy3-coupled goat anti-rabbit antiserum and Alexa fluor 488-coupled goat anti-mouse antiserum (1:200 dilution, Invitrogen) overnight at 4°C. Nuclei were stained by DAPI (1:10000 dilution, Sigma Aldrich). Slides were washed with 1xPTW for 4x5mins and mounted in Prolong Gold antifade reagent (Invitrogen Cat No. P36934).

### TUNEL staining

Mtz- and DMSO-treated adult *Platynereis* (45-55 segmented) worms were anesthetized and decapitated after ~28 hours treatment. The heads were fixed and mounted in Tissue Tek compound as described in the cryosectioning protocol. The TUNEL apoptotic cell death assay was performed by using the *In Situ* Cell death Detection Kit, TMR Red (Roche Cat No. 12156792910) immediately after sectioning without storage. The sections were fixed with 4% PFA/PBS for 20 min and equilibrated in 1xPBS for 30 min. Slides were incubated in freshly prepared permeabilization solution (0.1% Triton X-100 in 0.1% Sodium citrate) for 2 mins on ice. Slides were washed immediately with 1xPBS twice and re-suspended in TUNEL reaction mixture for 60mins at 37°C in a dark, humidified chamber. After reaction, the slides were washed in 1xPBS for 3x5 min and mounted in Prolong Gold antifade reagent (Invitrogen Cat No. P36934).

### Microscopy

For live imaging, premature adult worms of the desired genotype were anesthetized in 50:50 mixture of 7.5M MgCl_2_: natural seawater (NSW) and mounted on glass slides. Worms were covered with the same MgCl_2_:NSW mixture. Several layers of sticky tape (Tesa) served as spacer to avoid squashing. The eGFP expression in live worms was captured with a Zeiss Axioplan2 microscope.

Head images were taken with a 20x air objective, the tail images with a 40x oil immersion objective. Confocal images for sections were taken using a Zeiss LSM 710 confocal microscope with 405, 488 and 561-nm excitation wavelength. Sections were mounted in Prolong Gold antifade reagent. Images were processed using the ImageJ software package (http://imagej.nih.gov/ij/), to generate maximum intensity z-projections of scans.

## Results and Discussion

### Nitroreductase and egfp are specifically co-expressed under the r-opsin1 enhancer

We first generated an expression construct that should allow us to both visualize and ablate the cells we aimed to study. For this, we combined *egfp* and *ntr* via the ribosomal skip site *f2a* and placed this expression construct under the control of the already characterized *r-opsin1* enhancer ( [11], Figure 1A). Ribosomal skip sites are sequence stretches of viral origin that lead to the generation of separate proteins from a single mRNA [15]. This technique should result in equal amounts of both proteins, while minimizing the risk of mutual functional impairment, as it could arise from fusion proteins [15].

This construct was used to generate a *Platynereis* transgenic line driving reporter expression in the *r-opsin1*+ photoreceptors of the worm ( [11], Figure 1B). We next tested if the worms also expressed the coding sequence for *ntr*, and if this expression was also at similar levels as the *egfp* expression. For this, we performed whole mount in situ hybridization (WMISH) using antisense riboprobes against *ntr* and *egfp*. As expected, the spatial expression of *ntr* in transgenic worms was identical to *egfp* expression in the *r-opsin1*+ photoreceptors of the worm (Figure 1B-D). Furthermore, both stainings were similarly strong after the same amount of time, suggesting that equal amounts of *egfp* and *ntr* are produced, as predicted from the nature of the construct. These data demonstrate that *ntr* is faithfully co-expressed in the *r-opsin1*+ photoreceptors, as required for specific ablation of these cells.

### Metronidazole addition leads to a loss of fluorescently marked neurons and their projections

**Figure 2:**
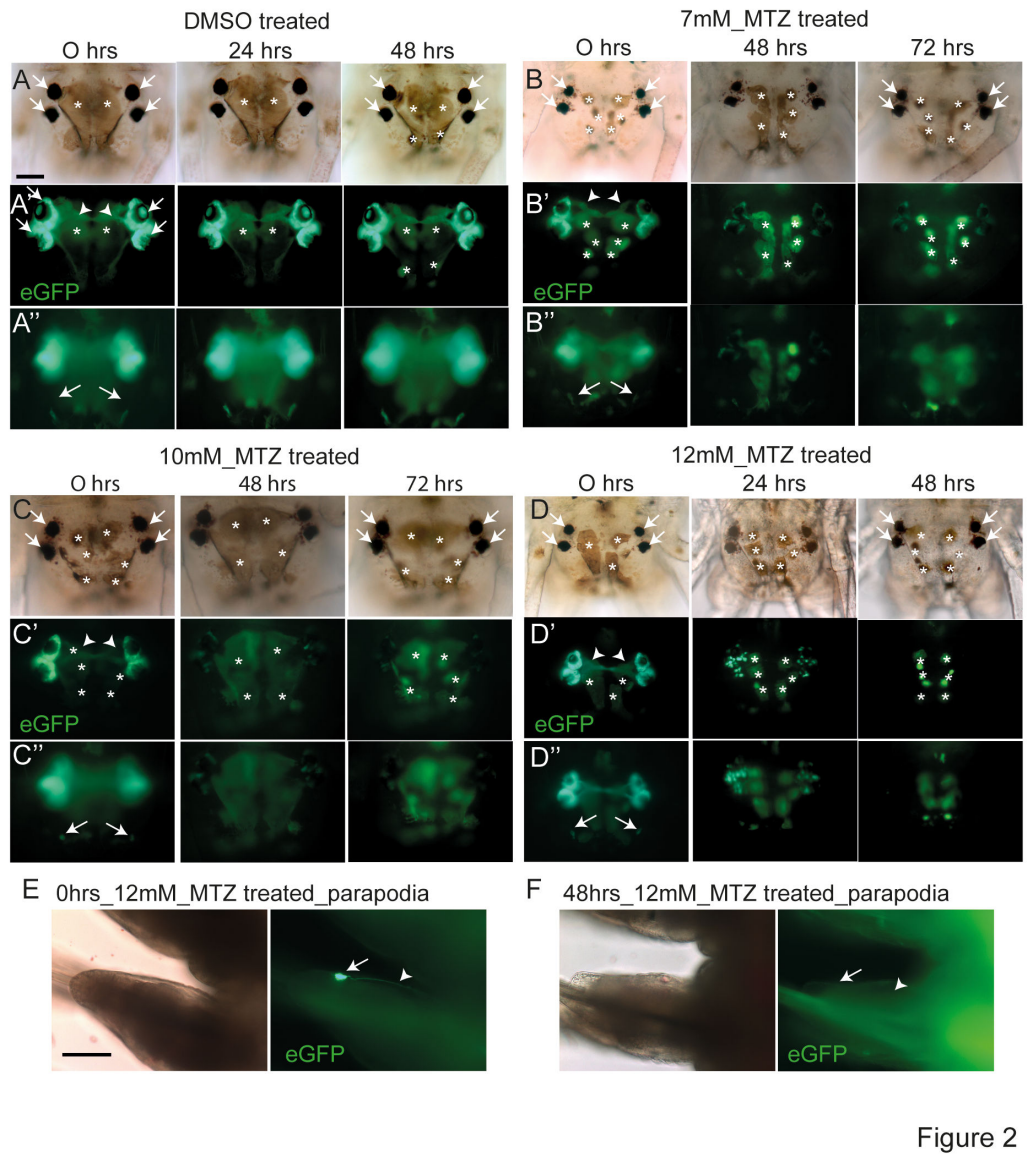
Metronidazole-dependent loss of eGFP fluorescence indicates an effect on *r-opsin1+* PRCs in transgenic animals. All images show live immature adult *r-opsin1::egfp-f2a-ntr* transgenic animals (10-20 segments). (A-D) Concentration- and incubation time-dependent loss of eGFP fluorescent cells from adult eyes and frontolateral eyelets. Animals were treated with DMSO (A-A”) or varying concentrations of metronidazole (B-B”: 7mM, C-C”: 10mM; D-D”: 12mM). Duration and type of treatment are indicated on top of each panel. Arrowheads point at the axonal projection from the adult eye photoreceptors. Asterisks: head pigment cells that show autofluorescence in the channel used for eGFP documentation. These pigment cells expand or contract depending on light intensity and time of day. Arrows indicate adult eye pigments (panels A-D) position of eGFP-expressing PRCs (panel A’), position of lateral frontal eyelets (A”-D”). (E,F) Loss of eGFP fluorescent non-cephalic PRC upon metronidazole treatment. (E) *Parapodium* with eGFP-expressing non-cephalic PRC (arrow) and its axonal projection (arrowhead) before metronidazole treatment; (F) same specimen after 48 hrs of exposure to 12mM mtz, with arrow and arrowhead indicating the same positions. Scale bars: 50µm.

If mtz was able to ablate transgenically labeled cells, we reasoned that exposure to the prodrug would reduce the number of eGFP-fluorescent PRCs in our *r-opsin1::egfp-f2a-ntr* strain. In a first series of experiments, we therefore incubated 10-20 segmented transgenic worms with three different concentrations of mtz in seawater (7mM, 10mM and 12mM). After 24hrs, 48hrs and 72hrs, we transferred the worms to plain seawater and checked for the pattern and cellular morphology of eGFP+ cells. DMSO-treated control worms never showed any effects on the number and morphology of eGFP- fluorescent cells (Figure 2A-A’’, see Figure 5G,H; S1D for quantification). In contrast, mtz treated animals showed a clear reduction of eGFP-fluorescent cells. After 24hrs, eGFP fluorescent cells in worms incubated with 12mM mtz were severely reduced in numbers (Figure 2D’), no projections of the adult eyes were visible (Figure 2D’) and fluorescence from the region of the lateral frontal eyelets was partly missing (Figure 2D’’). Worms incubated with lower concentrations of mtz (7mM and 10mM), but for extended times (48hrs / 72hrs), exhibited similar partial ablations (Figure 2B-B”, C-C”): Incubation with 7mM mtz for 48hrs or 72hrs led to a significant loss of fluorescent cells in the adult eyes (Figure 2B’), whereas the lateral frontal eyelets were always still visible (Figure 2B”); incubation with 10mM mtz for for 48hrs or 72hrs led to the loss of most fluorescent cells in the adult eyes (Figure 2C’) and of the lateral frontal eyelets (Figure 2C”). As expected, when exposure to the highest dosage (12mM mtz) was also extended to 48hrs, the treated worms lacked all eGFP-fluorescent cells. This included not only the photoreceptors in the head (Figure 2D’,D″), but also the non-cephalic photoreceptors of the trunk (arrows in Figure 2E,F). The neuronal projections of the investigated photoreceptors were absent in worms after any analyzed treatment (arrowheads in Figure 2A’-D’,E, F). Together, these data show that eGFP fluorescence is lost after mtz treatment in a concentration- and incubation time-dependent manner, consistent with the notion that the eGFP+ cells were successfully ablated.

While testing different mtz concentrations with worms of different sizes, we noted that the same concentration of mtz can differ in its efficiency depending on the size, i.e. number of segments, of the worm. Worms of the same absolute age can exhibit a range of different sizes, depending on the availability of food and animal density in the box. The size of the worm, best quantified by segment number, but not the absolute age, also determines when a worm will reach maturity [16]. We reasoned that the thickness of the cuticle and body wall will increase with segment number, and hence require a higher concentration of prodrug for penetration. Similarly to the aforementioned studies, we therefore carefully determined the most effective mtz concentrations (leading to the complete absence of GFP+ cells) for worms of different size categories. From these experiments, we suggest the use of the following mtz concentrations:

10-20 segmented worms: 12mM mtz
25-35 segmented worms: 17mM mtz
45-55 segmented worms: 25mM mtz

We also noted that worms homozygous for the transgene tended to respond more efficiently than worms that were only heterozygous, compatible with the possibility that the amount of *nitroreductase* expressed in the cells is a limiting factor in the ablation procedure. The concentrations we determined here work well for both worms that are homo- or heterozygous for the transgene.

### Metronidazole induces apoptosis in nitroreductase expressing cells

**Figure 3:**
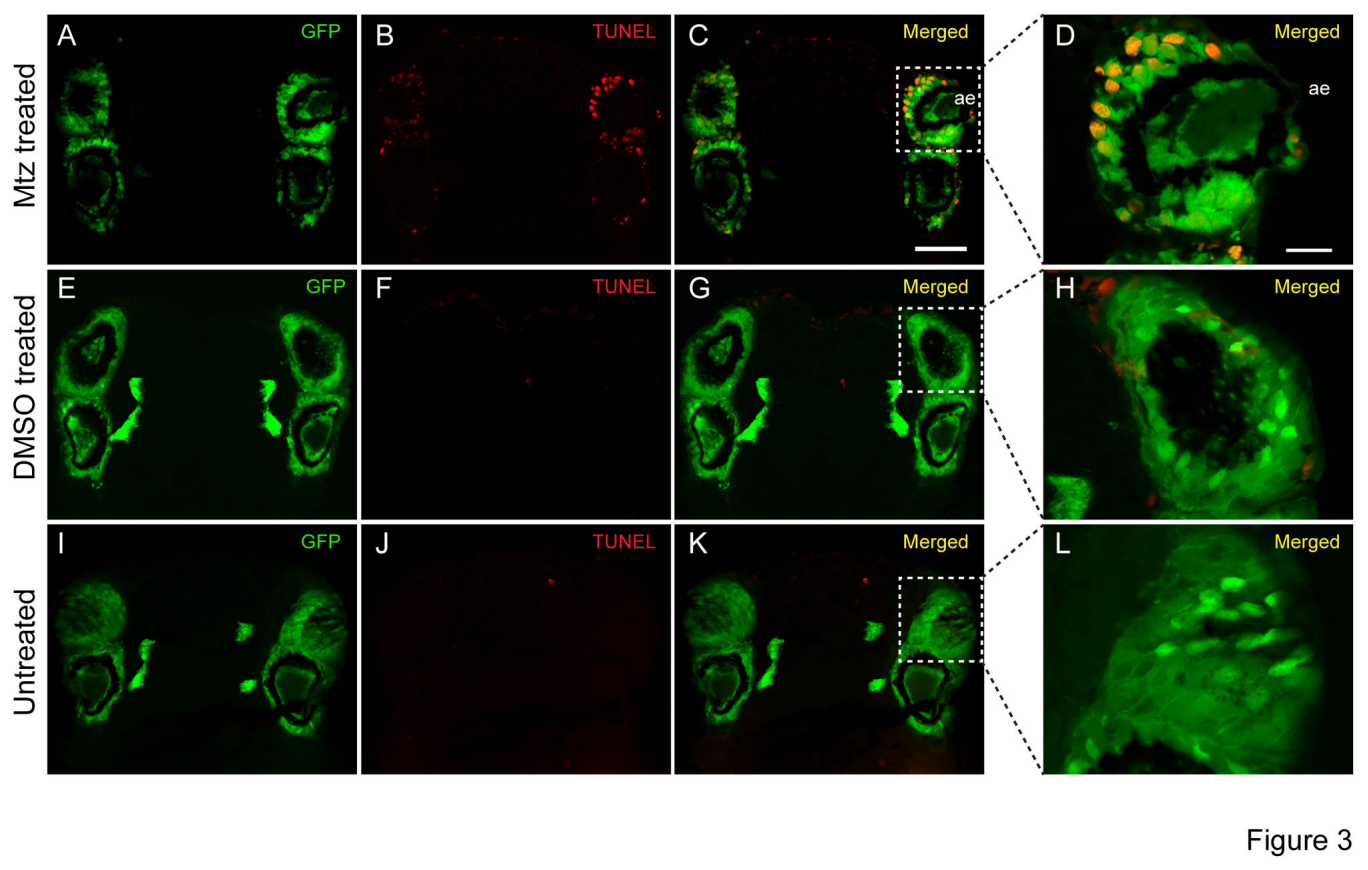
Metronidazole induces apoptosis in transgenically labeled cells. (A-L) Head sections of mtz treated (A-D), DMSO treated (E-H) and untreated (I-L) premature adult *r-opsin1::eGFP-f2A-ntr* worms (eye PRCs and projections, green) processed for terminal deoxynucleotidyl transferase-mediated deoxyuridinetriphosphate nick end-labelling (TUNEL) detection (red). (A-D) Apoptosis was detected in PRCs exposed to 25mM mtz after 28 hrs incubation, whereas transgenic animals treated with DMSO alone (E-H) or transgenic untreated animals (I-L), did not show staining above background. ae: adult eye. Scale bars C: 50µm; D: 15µm.

To verify that the loss of eGFP fluorescence was a result of the ablation of transgenically labeled cells, and not an effect of mtz on the properties of eGFP, we next tested if the addition of mtz leads to DNA fragmentation and subsequently to induced cell death. The free ends of DNA fragmented in the process of programmed cell death can be detected by terminal deoxynucleotidyl transferase–mediated deoxyuridinetriphosphate nick end-labeling (TUNEL) staining. We therefore established TUNEL detection in premature adult *Platynereisdumerilii* specimens (45-55 segmented worms). In accordance with the determined optimal concentrations of mtz, we treated worms with 25mM mtz. When analyzed after 28hrs, a well-detectable TUNEL staining was detectable in the areas of eGFP expression, such as the photoreceptors of the adult eyes (Figure 3A-D). Untreated transgenic siblings or addition of DMSO did not show any increase in TUNEL staining over background (Figure 3E-L). These data are in agreement with the idea that ntr-positive cells convert mtz to a cytotoxic compound that then leads to apoptotic death of these cells. Our conditions were highly similar to the treatments used in zebrafish [8,17].

**Figure 4:**
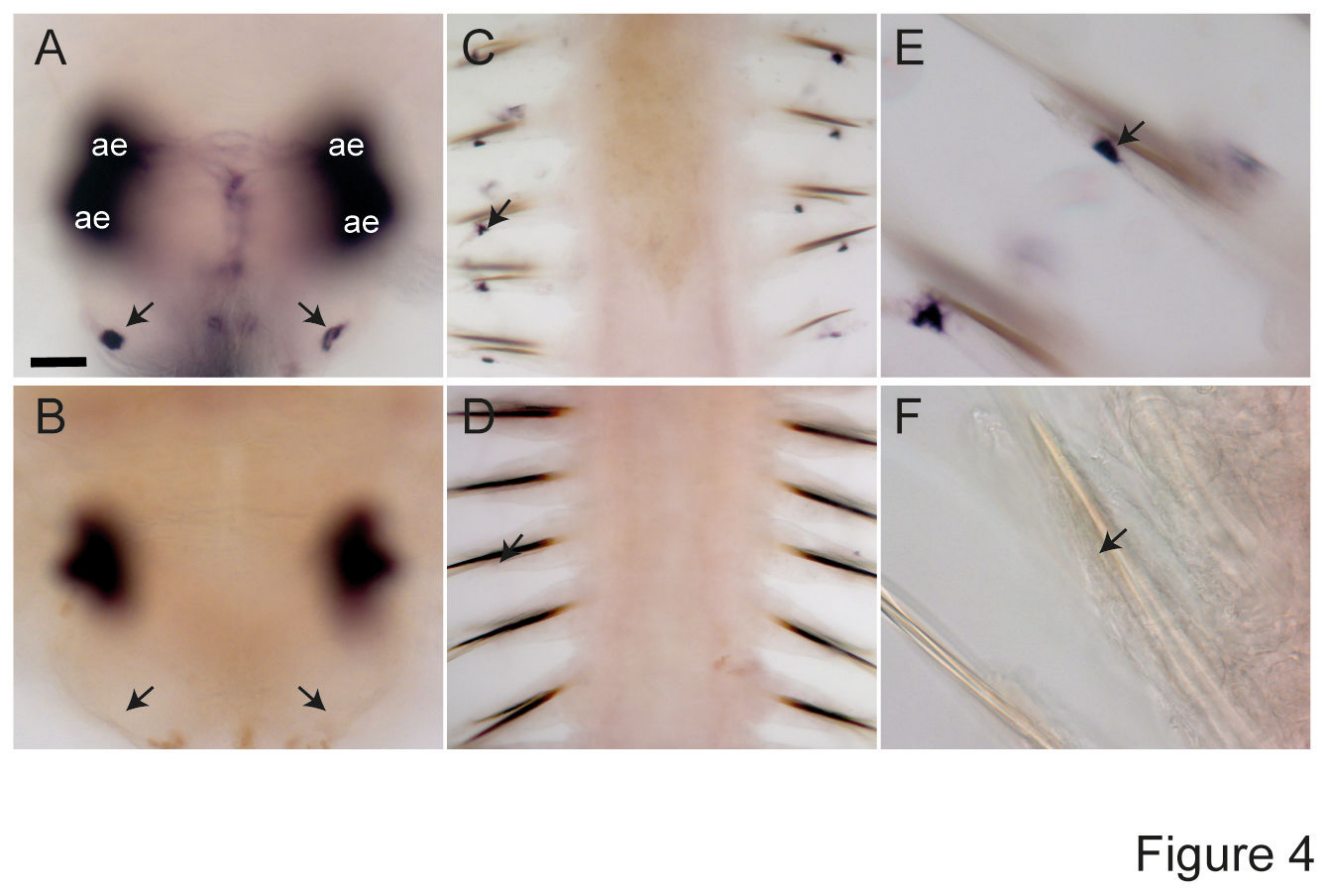
Loss of *r-opsin1+* cells by metronidazole treatment. Whole mount *in* *situ* hybridization with a riboprobe specific for *Platynereis* *r-opsin1* on immature adult *Platynereis* worms (10-20 segments). (A,C,E) DMSO controls, (B,D,F) metronidazole treated worms (12mM, 48hrs). (A,B) Dorsal views on heads (anterior down), focused on the position of the lateral frontal eyelets (arrows). (C-F) Ventral views (anterior down) on immature adult worm tails (C,E) treated with DMSO, (D,F) treated with mtz. Arrows point at the position of the peripheral *r-opsin1+* photoreceptor cells. Black ‘needle-like’ structures in C-F are aciculae (bristles). Scale bar: 30µm.

**Figure 5:**
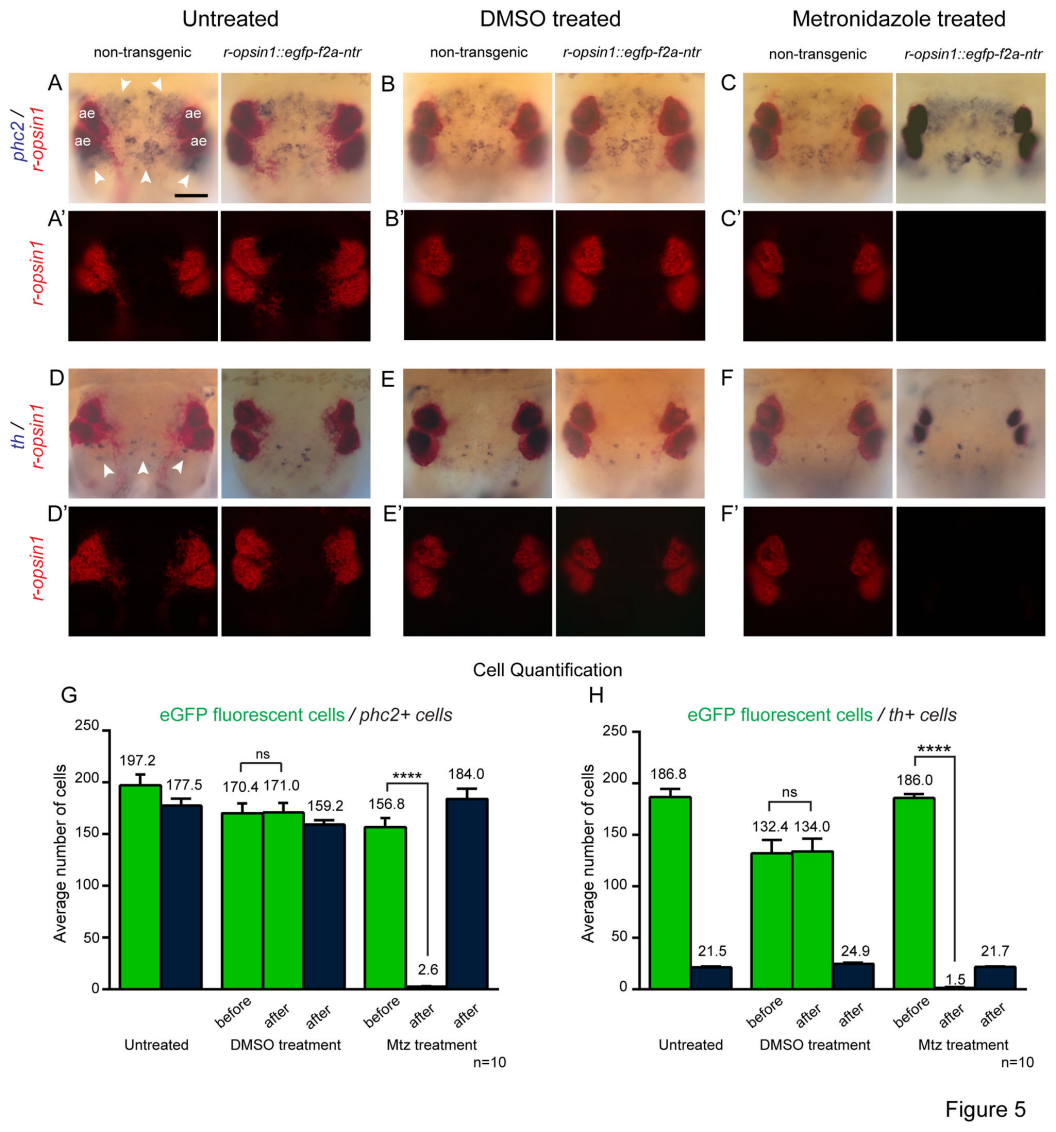
Metronidazole treatment specifically ablates *ntr* expressing cells, without affecting other brain cells. (A-F) Metronidazole treatment has no effect on non-PRC marker genes. Comparative analysis of expression patterns of the neuronal marker genes *prohormone* *convertase2/phc2* (arrowheads in A-C) and *tyrosine* *hydroxylase/th* (arrowheads in D-F) in untreated (A,A’,D,D’), DMSO treated (B,B’,E,E’) and metronidazole treated (C,C’,F,F’) animals. Each set of panels compares non-transgenic control animals (left) and *r-opsin1::eGFP-f2A-ntr* transgenic animals. Neuronal marker genes are detected in blue, *r-opsin1* were detected with FastRed substrate (red). In panels (A’-F’), FastRed is visualized using fluorescence microscopy. Scale bar: 50µm. (G,H) Quantification of cell numbers in untreated, DMSO treated and mtz treated animals. Individual eGFP fluorescent PRCs (green bars) were counted in live transgenic worms (same animals were counted before and after treatment). *phc2* (G) and *th* (H) expressing cells (black bars) were determined by counting all cells that showed complete cellular outlines in WMISH analyses of animals fixed after the respective experiment. Data represent means ± S.E.M. (n=10 worms for each experiment). ****p<0.0001; ns. - no statistically significant differences. The two-tailed paired student *t*-test was used for statistical analyses.

In order to validate that mtz treatment indeed depleted the number of photoreceptors, we performed whole mount in situ hybridization (WMISH) using a riboprobe against *Platynereis ropsin1* endogenously expressed in the transgenically labeled cells [11]. Worms with 10-20 segments, incubated in 12mM mtz for 48hrs, showed a complete loss of the WMISH signal at the position of the lateral frontal eyelets in the head (arrows in Figure 4A,B), as well as in the worm’s tail (arrows in Figure 4C-F). As the dark blue NBT/BCIP precipitate is difficult to discriminate from the brown/black eye pigment present in the adult eyes, we used a different detection precipitate (Fast Red) to assess *r-opsin1* expression in the adult eyes. No *r-opsin1* mRNA can be detected in the adult eyes after mtz treatment (17mM mtz based on segment number) of *r-opsin1::egfp-f2a-ntr* animals (Figure 5A-F, A’-F’; S1A-C,A’-C’). In contrast, untreated or DMSO-treated transgenic siblings, as well as mtz-treated non-transgenic animals showed no reduction in staining (red staining in Figure 5A-F, A’-F’; S1A-C,A’-C’).

Taken together, the analyses of two independent markers – eGFP fluorescence and *r-opsin1* mRNA expression – along with the presence of a TUNEL signal, provide strong evidence that mtz incubation leads to apoptosis of *nitroreductase* expressing cells in *Platynereis*.

### Metronidazole-mediated ablation is restricted to nitroreductase expressing cells

In order to assess the specificity of cell ablation in *Platynereis*, we next investigated if cell ablation was restricted to the *ntr* expressing cells, or caused more general defects. We tested this by three independent approaches.

First, we focused again on the large adult eyes of *Platynereis*. In these eyes, *r-opsin1*+ photoreceptor cells are directly adjacent to pigment cells [18]. If the cell ablation is specific, these directly neighboring pigment cells should remain unaffected. This was indeed observed when we inspected the mtz-treated animals. The pigment cells remained present and were indistinguishable from the pigment cells of all control worms (arrows Figure 2A,B,C,D).

In a second step, we aimed at quantifying the effect of mtz on GFP+ (and hence NTR+) versus GFP- (and hence NTR-) cells. Such quantification is difficult to perform with the pigment cells of the adult eyes, as they are very densely packed and thus difficult to count reliably. Therefore, we performed WMISH with three different genes, whose mRNA is present in cells located in different areas in the head, but not in the *ropsin1+* photoreceptors. We visualized the cells expressing *Pdu-prohormone convertase 2* (*phc2*) a prohormone processing enzyme, demarcating neurosecretory cells [3], *Pdu-brn1/2/4* a pou-class transcription factor [2], as well as *Pdu- tyrosine hydroxylase* (*th*) [2], the enzyme catalyzing L-dopa synthesis [19] with the blue NBT/BCIP precipitate. We also counter-stained for *r-opsin1* mRNA in the same animals with the red Fast Red substrate (red in Figure 5A-F,A’-F’, S1A-C,A’-C’). As expected, *r-opsin1* was rarely detectable or undetectable in mtz treated animals compared to untreated and DMSO treated controls (Figures 5A’-F’, S1A’-C’). In contrast, *phc2*, *brn1/2/4* and *th* staining was indistinguishable in mtz treated vs. untreated or DMSO treated animals (blue in Figure 5A-F; S1A-C). This observation was further substantiated by quantification: Whereas there was no significant difference in the number of GFP+ cells in untreated or DMSO treated animals, this number dropped to nearly undetectable in mtz treated animals (green bars in Figure 5G,H; S1D). By comparison, the cell numbers of *phc2*, *brn1/2/4* and *th* (please note that *th+* cells are present at different focal levels) showed no such difference in any of the treatments (black bars in Figure 5G,H;S1D).

**Figure 6:**
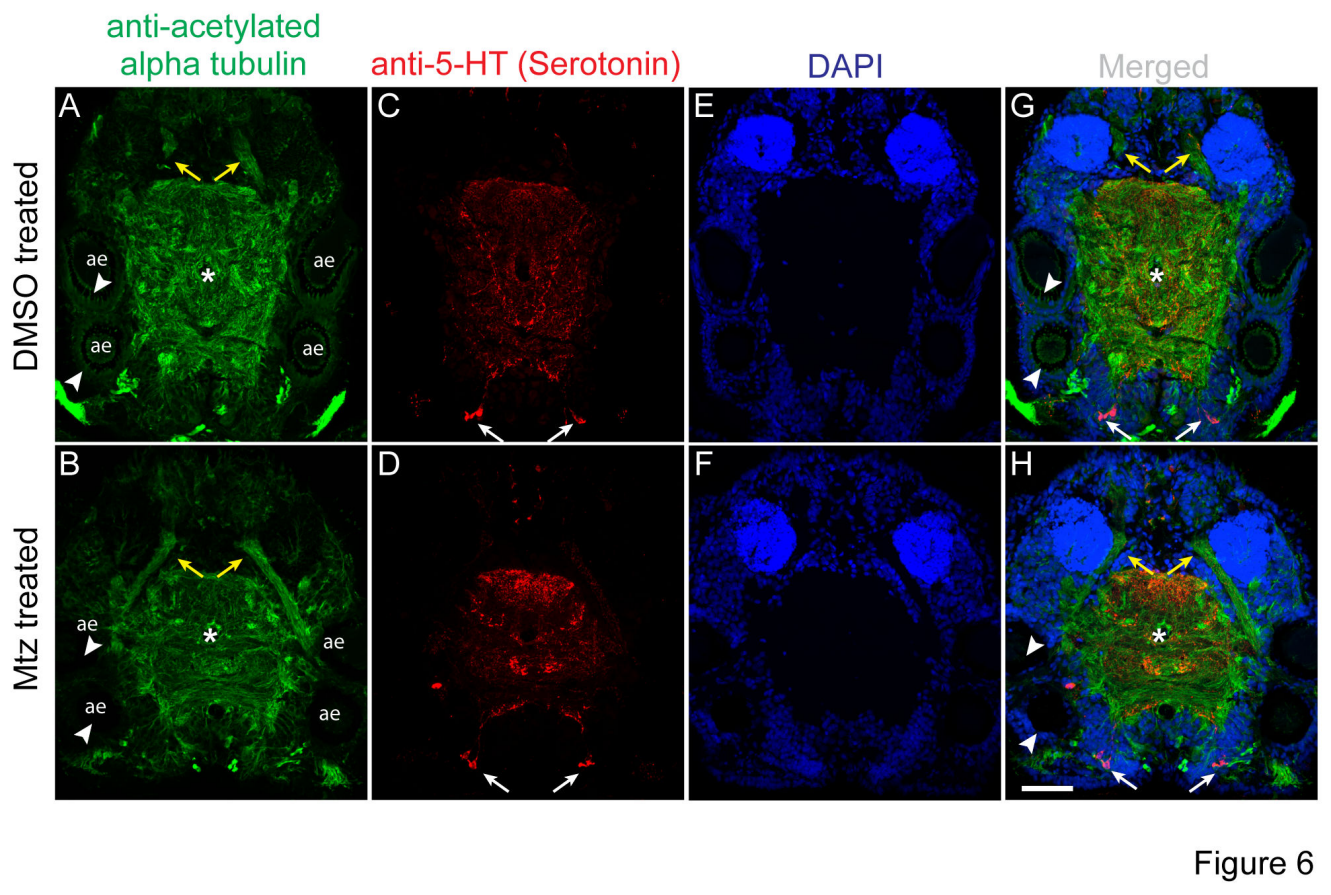
Metronidazole treatment does not affect general brain anatomy of *Platynereisdumerilii* . Confocal images of 10µm thick horizontal cryosections from premature adult transgenic *Platynereis* heads (r-opsin1::egfp-f2a-ntr), stained with antibodies against acetylated tubulin (A,B; green), and serotonin (C,D; red), as well as with the DNA-binding agent DAPI (E,F; blue). Panels G and H are merged views of all three channels. Animals (45-55 segments) were treated with DMSO (A,C,E,G) or 25mM metronidazole (B,D,F,H) for ~72-80hrs. No morphological differences were observed in the overall neuropil structure and wiring (B,H, compare to A, G) and the position and wiring of serotonergic neurons (D,H, compare to C, G) in ablated animals compared to controls. asterisks: center of neuropil, arrows: posterior serotonergic neuron cell bodies, yellow arrowheads: antennal nerve, ae: position of adult eyes; white arrowheads: position of GFP+ PRCs in control animals (A,G) and equivalent position in metronidazole-treated animals (B,H) Note absence of eGFP fluorescence in B,H, compared to A ,G, indicative of the successful ablation of the *r-opsin1+* PRCs. Scale bar: 50µm; dorsal views, anterior up. Also compare to reference [2] for details of the brain scaffold.

Third, we assessed, if the axonal and dendritic scaffold of the worms is affected by the mtz treatment. Neurite connections are highly stereotypic and have thus been used as alignment grid for the comparison of expression patterns [2-4]. If mtz treatment induced general tissue damage, we reasoned that this should be detectable in disruptions or alterations of the neurite scaffold. In order to test this, we visualized the overall neuronal scaffold using an antibody directed against acetylated tubulin [2-4]. We also used an antibody directed against serotonin, to more specifically investigate a subset of the worm’s axons [3]. Furthermore, we visualized all cell nuclei by DAPI staining, which should reveal any larger aberrations in the brains of mtz treated worms compared to controls. When comparing the overall neurite scaffold (Figure 6A,B,G,H, arrowheads point at the position of GFP expressing PRCs), as well as the axon tracts and cell bodies of serotonergic neurons (Figure 6C,D,G,H) of mtz treated and control worms, we did not observe any general alterations in mtz treated animals indicative of unspecific tissue damage. Similarly, we did not note any difference in overall position of the neuron and glia cells in mtz vs. DMSO treated control worms by the comparison of DAPI stained heads (Figure 6E-H).

Taken together, these data strongly suggest that NTR/mtz-mediated cell ablations are confined to the cells expressing *ntr*, and can therefore be used as tool to study specific cell functions in *Platynereisdumerilii* .

### Metronidazole-induced specific cell ablation as tool for cell type analyses in *Platynereis dumerilii*

Here we report on the establishment of a technique to specifically ablate cell types in the marine bristle worm *Platynereisdumerilii* . This technique now provides a tool to test the function of cell types that were previously not accessible. As the system is inducible, any given cell type can first function normally during the development of the animals, excluding indirect developmental defects prior to the time of assay. Currently, the major limitation for its use in *Platynereisdumerilii* is the availability of cell type specific enhancer constructs.

Whereas mtz- mediated cell ablation has been used in well-established molecular model systems, most prominently in zebrafish, with great success, its use in less conventional molecular animal systems – common in the field of evolutionary development – has so far not been explored. We therefore anticipate that this study will also be useful for the establishment of mtz-mediated cell ablation in other model systems. From our establishment of the system in *Platynereisdumerilii* , we noted two main points that are likely of general relevance for the usage of this technique in other animal systems. First, we noted a dependence of the effectiveness of the treatment on the age (size) of the animals, and likely also on the expression levels of *nitroreductase* within the targeted cells. This suggests that the exact concentrations and incubation time with mtz should be cross-checked for different animal stages and new expression constructs. Secondly, it has recently been suggested that mtz can induce general DNA damage in *Drosophila* cells [20]. The respective controls of mtz-treated non-transgenic animals will, of course be required to rule out possible unspecific effects in any phenotypic assay. However, our extensive analyses show that at the used concentrations, mtz neither leads to elevated apoptosis nor to general aberrations in brain morphology, or change in neuronal marker genes expressed outside the *ntr* expressing cell population. This makes mtz induced ablation of transgenically *ntr* expressing cells a useful tool to obtain a functional understanding of *Platynereisdumerilii* cell types.

## Supporting Information

Figure S1 **Expression of *brn1/2/4* in control vs. mtz-treated animals.** Related to Figure 5.
(A-C) WMISH of *brn1/2/4* (blue), counterstained with *r-opsin1* (red), white dotted circle indicates major expression domain of *brn1/2/4*, used for quantification. (A’-C’) Fluorescent visualization of the Fast Red precipitate, used for *r-opsin1* expression detection. Dorsal views, anterior down
Blue staining at the very anterior of the head (out of focus in A) is unspecific background staining caused by probe trapping in gland cells. (D) Quantification of cell numbers in mtz- treated vs. control worms. Green bars: number of GFP+ cells in live animals (same animals were counted before and after treatment). Black bars: number of *brn1/2/4* cells in fixed animals (can only be counted once).
Details of treatment conditions and quantification as described for Figure 5. Data represent means ± S.E.M. (n=10 for each experiment). ****p<0.0001; ns. - no statistically significant difference. The two-tailed paired Student *t*-test was used for statistical analyses. Scale bar: 50µm.
(TIF)
