# Supplementary figures and images for: Conditional and Specific Cell Ablation in the Marine Annelid Platynereis dumerilii
Source: PLoS One. 2013 Sep 24;8(9):e75811. doi: 10.1371/journal.pone.0075811 (PMC3782428; doi:10.1371/journal.pone.0075811)

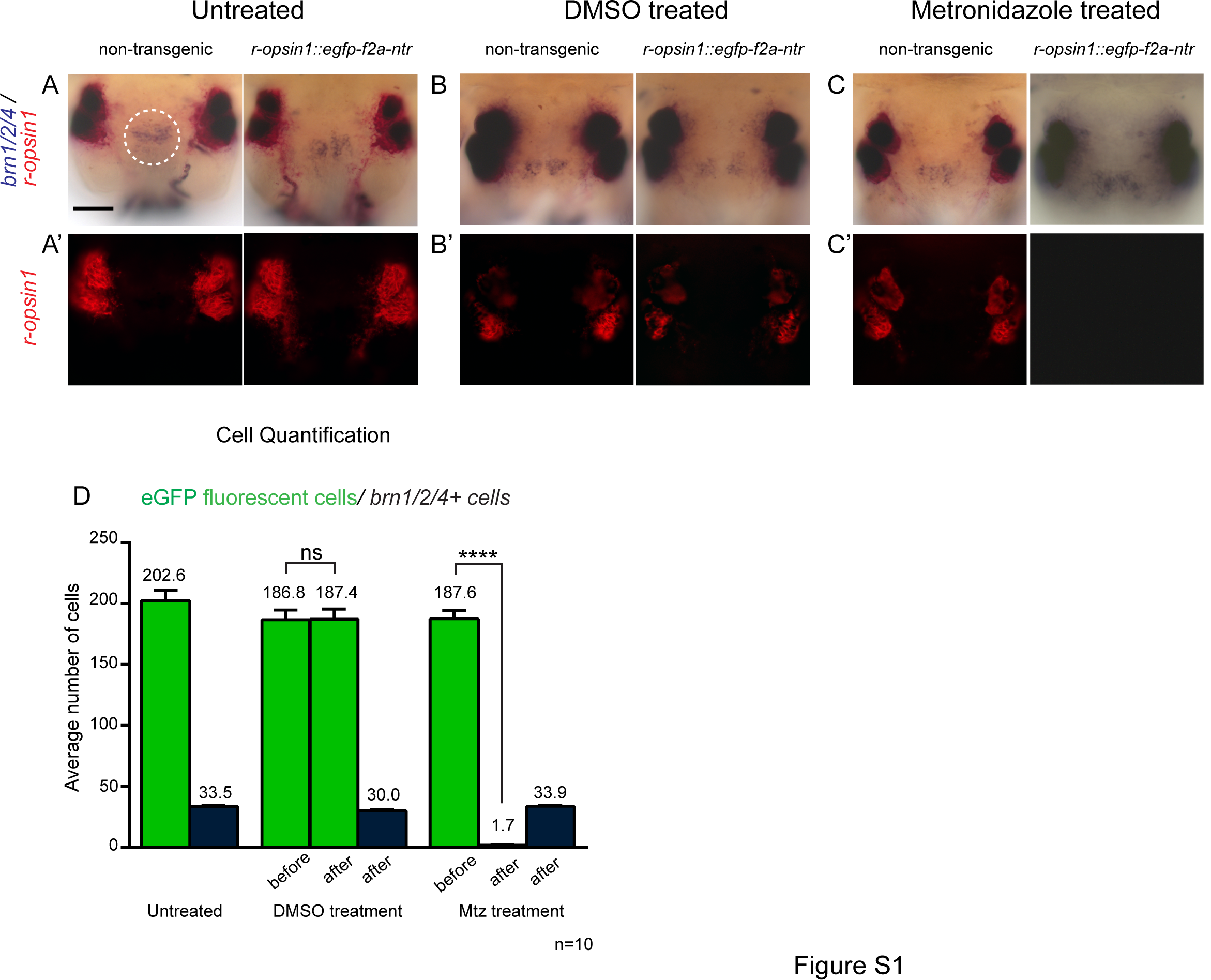

Supplement: Figure S1 — Expression of brn1/2/4 in control vs. mtz-treated animals. Related to Figure 5. (A-C) WMISH of brn1/2/4 (blue), counterstained with r-opsin1 (red), white dotted circle indicates major expression domain of brn1/2/4, used for quantification. (A’-C’) Fluorescent visualization of the Fast Red precipitate, used for r-opsin1 expression detection. Dorsal views, anterior down Blue staining at the very anterior of the head (out of focus in A) is unspecific background staining caused by probe trapping in gland cells. (D) Quantification of cell numbers in mtz- treated vs. control worms. Green bars: number of GFP+ cells in live animals (same animals were counted before and after treatment). Black bars: number of brn1/2/4 cells in fixed animals (can only be counted once). Details of treatment conditions and quantification as described for Figure 5. Data represent means ± S.E.M. (n=10 for each experiment). ****p<0.0001; ns. - no statistically significant difference. The two-tailed paired Student t-test was used for statistical analyses. Scale bar: 50µm. (TIF) [file pone.0075811.s001.tif]
